# The History and Capabilities of Herbal Simples

**Published:** 1892-08-13

**Authors:** W. T. Fernie


### The History and Capabilities of Herbal Simples.

By W. T. Fernie, M.D.

*(Continued from page 288, Vol. XI.)*

### THE COMMON FLAG.

Following an example set by true whigs of the old school our English land and water flags hang out their coloured banners of purple and yellow respectively, whilst deriving their generic name from this display of their petals like political bunting. Each is also called iris, as resembling the rainbow in beauty, and variegated hues. The land flag (Iris versicolor) is well known as growing in swamps and moist meadows, with sword-shaped leaves, and large purple heads of flowers bearing claws of petals chiefly dark blue, whilst veined with green, yellow, and white. The water flag (Iris pseudo-acorus) is similar in growth, and equally well known by its heads of brilliant yellow flowers, with blade-like leaves, in wet places and watercourses. Also the stinking iris (iris fœtidissimal) is a British species abundant in southern England, where it is found in woods and shady places. It is generally called stinking gladdon, or roast beef, because the leaves when bruised emit a strong smell of that viand. The term glad—or smooth—refers to the leaves, and the plant has flowers of a dull livid purple, smaller than those of the other flags. Lastly, there is the sweet flag (Acorus calamus), though this is not an iris, but belongs botanically to the family of arums. It grows on the edges of lakes and of streams all over Europe, as a highly aromatic, reedy plant with an erect flowering stem of yellowish-green colour. The root of the blue flag contains chemically an oleoresin which is purgative to the liver, and very useful for bilious sickness. A medicinal tincture is made from it by the chemists, which holds the iris in solution, and is admirably useful in an attack of bilious vomiting, with sick headache, and a film before the eyes. Three or four drops of the tincture may be taken directly, with a tablespoonful of cold water, and the same dose repeated in half an hour if necessary. It is a trustworthy substitute for calomel, or blue pill. Louis the Seventh, of France, chose this blue flag as his heraldic emblem; hence its name, "Fleur de lys," has been subsequently borne upon the arms of France. Some say the flower he chose was a white iris. In "Chaucer" we read: "his nekke whit' was as the flour de lys." The flower was figured on a shield sent down from heaven to King Louis, or Clovis, when fighting against the Saracens. This purple flag was formerly considered sacred to the Virgin. A knight more devout than learned could never remember beyond two words of the Latin prayer offered to the Holy Mother. These were "Ave Maria," repeated night and day until the good old man died. Then a plant of the blue iris sprang up on his grave, displaying in golden letters on every flower the words "Ave Maria." On the monks opening the tomb they found the root of the plant resting on the lips of the holy knight whose body lay buried below. The orris powder popular in the nursery, and with ladies for toilet purposes on account of its fresh violet scent, is made from the root of the iris, and has its name from the genitive-ireos. The yellow flag, or water flag, is called in the north, seggs. Its flowers afford a beautiful yellow dye. Its seeds when roasted can be used instead of coffee. The juice of the root is very acrid when sniffed up the nostrils, and causes a copious flow of water therefrom, which gives marked relief in cases of obstinate headache. In Argyleshire the root chopped up and chewed is said to be a cure for toothache. This plant is named levers in the South of England. The sweet flag (Acorus calamus) derives its first name from the Greek, *koree*, or pupil of the eye, because of its being used in diseases of that organ. Calamus was a Roman name for a reed. Formerly, by reason of its agreeable odour, like that of violets, the plant was freely strewn on the floors of cathedrals at times of church festivals, and in many private houses instead of rushes. Its root is a powerful cordial against flatulence and for the headache of cold indigestion. It contains a volatile oil and a bitter principle, acorin. A fluid extract is prepared by the chemists, of which from thirty to forty drops may be given for a dose every half-hour for several consecutive times. The candied root is much employed for like purposes in Turkey and India. It is sold as a favourite medicine in every Indian bazaar. Ainslie says it is reckoned so valuable in the bowel complaints of children that there is a penalty incurred by every druggist who will not open his door in the middle of the night to sell it if demanded. The root stocks are brought to this country from Germany, and are employed for flavouring beer, for making snuff, and for masticating to clear the urine.

